# Collaborative nursing practices supporting home-based living for people living with cancer and dementia: A modified grounded theory study

**DOI:** 10.1016/j.apjon.2026.101048

**Published:** 2026-10-17

**Authors:** Ayumi Takao, Ikumi Iida, Hiroko Sakuma, Hiromi Sakuda

**Affiliations:** a Osaka Metropolitan University Graduate School of Nursing, Abeno, Osaka, Japan; b Palliative Care Team, Department of Nursing, National Cancer Center Hospital, Chuo-ku, Tokyo, Japan; c Palliative Care Center, Kansai Medical University Hospital, Hirakata, Osaka, Japan

**Keywords:** Neoplasms, Dementia, Nursing, Collaboration, Comorbidity, Aged

## Abstract

**Objective:**

As the number of people living with cancer and dementia increases with population aging, effective collaboration between hospital-based and home-visiting nurses is essential to support meaningful home-based living. However, little is known about how nurses collaboratively manage the instability arising from the concurrent illness trajectories of cancer and dementia. This study aimed to elucidate the collaborative nursing practices that support home-based living for people living with cancer and dementia.

**Methods:**

Semi-structured interviews were conducted with 21 nurses working at cancer treatment hospitals or home-visiting nursing stations between May and December 2025. Data were analyzed using a modified grounded theory approach.

**Results:**

The core category was “Recalibrating life continuity while living with cancer and dementia,” which represented a case-centered and continuous process underlying all categories. The six categories were: (1) Team building for high-quality geriatric oncology nursing; (2) Reconstructing the patient's intention; (3) Reorganizing the care system to sustain home-based living; (4) Anticipating interactions between cancer- and dementia-related symptoms and sharing management strategies; (5) Preventing or mitigating functional decline with disease progression; and (6) Caring for families experiencing dementia-related ambiguous loss and cancer-related losses.

**Conclusions:**

The findings highlight case-centered collaboration across care settings as a dynamic process for addressing the complex vulnerabilities associated with the coexistence of cancer and dementia. The proposed model conceptualizes how nurses adapt collaborative practices in response to changing patient and family needs while orienting care toward continuity of life at home.

## Introduction

Cancer is an age-related disease, with approximately 60% of cases occurring in adults aged 65 years or older in developed countries.^1^ Dementia is also highly prevalent among older adults. Globally, an estimated 57.4 million people were living with dementia in 2019, and this figure is projected to reach 152.8 million by 2050.^2^ Consequently, the number of people living with both cancer and dementia is expected to increase. Previous studies have reported the presence of dementia in approximately 5% to 6% of people included in cancer registries and hospital-based studies.^3^, ^4^

People with cancer and dementia have complex health care needs and often experience inadequate symptom control.^5^, ^6^, ^7^ They are more likely to be diagnosed with advanced cancer and are unlikely to receive aggressive anticancer treatment.^8^, ^9^, ^10^ Furthermore, they have higher rates of hospitalization, emergency department visits, and readmissions than people with cancer who do not have dementia.^11^, ^12^ Additionally, receiving a cancer diagnosis can trigger psychological distress, particularly in people with cognitive impairment, including anxiety related to impaired decision-making and uncertainty about the future.^13^, ^14^, ^15^ These interacting physical, cognitive, and psychological challenges may exacerbate dementia-related symptoms, including behavioral and psychological symptoms of dementia (BPSD), and lead to variations in daily functioning and care needs.

People with dementia are vulnerable to influences from their care environment, and being able to remain safely in a familiar place is an important factor affecting their quality of life.^16^, ^17^, ^18^ However, a previous study examining the achievement of a good death among people with cancer and dementia reported that overall quality of death was consistent irrespective of the presence or absence of dementia. Nevertheless, people with both cancer and dementia showed a lower level of achievement for the item “patient was able to stay at his or her favorite place.”^19^ These findings suggest that supporting home-based living for people with both cancer and dementia is challenging due to the instability associated with concurrent illness.

Supporting home-based living for people with both cancer and dementia requires careful and responsive nursing care from a life-oriented perspective, given the complexity and uncertainty of their conditions. Previous studies have described challenges in decision-making and cancer care for people living with dementia, highlighting the need for repeated assessment, individualized and flexible care based on a holistic understanding of the individual, and appropriate training for health care professionals.^20^, ^21^, ^22^, ^23^ However, these studies have focused primarily on decision-making and care within cancer treatment settings. In particular, it remains unclear how hospital and home-visiting nurses collaborate to manage the instability arising from the coexistence of cancer and dementia while supporting continued home-based living. Therefore, this study aimed to elucidate the collaborative nursing practices that support home-based living for people living with cancer and dementia.

## Methods

### Study design

This qualitative study employed the Modified Grounded Theory Approach (M-GTA)^24^ using semi-structured interviews. M-GTA generates explanatory concepts and a conceptual framework by examining relationships among concepts within participants' narratives. Because it focuses on dynamic social interactions, it was appropriate for exploring collaborative nursing practices between hospital and home-visiting nurses who support home-based living for people living with cancer and dementia.

### Participants and recruitment

Participant recruitment initially followed purposive sampling based on predefined inclusion criteria. Initial participants were identified through the co-authors’ professional network in oncology nursing, followed by snowball sampling to recruit additional participants. The referring individuals first confirmed the willingness of potential participants to receive study information. Interested nurses were then contacted by the first or third author via e-mail and provided with detailed study materials. Once the candidates expressed initial interest, administrative permission to conduct the study was obtained from their respective affiliated institutions. After securing institutional clearance, the first author arranged a meeting with each participant to address any questions, ensure adequate time for consideration, and obtain written informed consent prior to the interview.

Because data collection and analysis were conducted concurrently in accordance with the M-GTA, participant recruitment was subsequently guided by the ongoing analysis in accordance with the principles of theoretical sampling. Emerging concepts guided the selection of subsequent participants. For example, when early interviews with hospital nurses generated concepts regarding differences in care goals and values between hospital and home-care settings, subsequent participants were recruited from home-visiting nursing stations to explore whether similar experiences existed and how these differences were addressed.

The inclusion criteria were as follows:

Nurses with at least five years of clinical nursing experience.

Nurses who had prior experience supporting home-based living for people with cancer and dementia.

Individuals capable of communicating in Japanese.

### Data collection

A semi-structured interview was conducted with each participant between May and December 2025. Interviews were conducted either online or in person, depending on participant preference. All interviews were conducted by the first author (AT), a certified nurse specialist in oncology nursing with clinical and qualitative research experience, and were audio-recorded with the participants' consent. The interviewer had no prior clinical or supervisory relationship with the participants. The first author's clinical experience in oncology nursing facilitated the use of probing questions to explore participants' experiences in greater depth while remaining aware of the potential influence of prior assumptions. The interview guide (Supplementary Table S1) comprised broad, open-ended questions covering (1) challenges experienced by people with cancer and dementia and their families in continuing home-based living from the time of cancer diagnosis through the end of life; (2) nursing care provided when they or their families encountered these difficulties; and (3) collaborative practice between cancer treatment hospitals and home health care services in supporting home-based living for people living with cancer and dementia. It remained unchanged throughout the study, while additional probing questions were used to explore emerging concepts based on participants' responses. In addition, demographic information, including participants' age, gender, institutional affiliation, years of clinical experience, the number of people living with cancer and dementia they had cared for in the past year, and whether they held specialized certifications, was collected. The interviews lasted 48–90 minutes, with a mean duration of 62 minutes.

### Data analysis

Data were analyzed using the Modified Grounded Theory Approach (M-GTA).^24^ Analysis focused on the contextual meanings of participants' narratives while considering their perceptions, actions, and situational factors.

1. All interviews were transcribed verbatim into Word documents. The verbatim transcripts were read repeatedly.
2. We used an analytical worksheet comprising “Variation,” “Theoretical notes,” “Definition of concept,” and “Concept name.” The analytical focus was defined as “nurses supporting home-based living for people living with cancer and dementia.” The analytical theme was “What constitutes collaborative practices to support home-based living for people living with cancer and dementia?” Based on the first data sample, descriptions related to practices were entered into the “Variation” column. The reasons for selecting the data and its interpretation were recorded in the “Theoretical notes” column.
3. After generating concepts from the first data sample, we applied the same analytical procedure to subsequent samples. We used constant comparative analysis to examine similarities and differences across cases.
4. Concepts were defined and named in a manner consistent with the data so that the meaning of each extracted variation was preserved. Analysis was repeated until all similar concrete examples were identified.
5. In parallel with concept generation, we developed subcategories to represent the relationships among concepts, eventually forming broader categories.
6. We created a figure illustrating the relationships among subcategories and categories, which were integrated into a single core category.
7. After analyzing data from the first 16 participants, the major categories and a preliminary conceptual framework had been established. At this stage, the ongoing analysis indicated that decision-making support and multidisciplinary collaboration required further elaboration, whereas practices related to maintaining physical function had been less fully developed. The final five participants comprised three generalist home-visiting nurses and two hospital nurses (one certified nurse in dementia nursing and one certified nurse specialist in oncology nursing), who were recruited to further examine these developing aspects of the conceptual framework. The additional interviews confirmed the developing conceptual framework, particularly regarding decision-making support and multidisciplinary collaboration, while providing little additional variation in practices related to maintaining physical function. No new concepts, subcategories, categories, or relationships requiring modification of the framework emerged. The research team therefore concluded that the conceptual framework sufficiently explained the study phenomenon and that theoretical saturation had been achieved after analysis of all 21 participants.
8. The primary analysis was conducted by the first author (AT). The developing concepts, subcategories, categories, and their relationships were reviewed by the third author (HS) using the interview data. The analysis was refined through ongoing discussions between the first and third authors, with additional supervision from the fourth author (HSa) and, when appropriate, the second author (II). Any differences in interpretation were resolved through discussion until consensus was reached. The researchers recognized that their oncology nursing experience and prior assumptions could influence data interpretation and therefore documented their reflections in theoretical notes and analytical memos throughout the analysis to critically examine emerging interpretations. The final conceptual framework was synthesized into a storyline. This study was reported in accordance with the Standards for Reporting Qualitative Research (SRQR) guidelines.^25^

### Trustworthiness

Trustworthiness of the analysis was ensured according to Lincoln and Guba's criteria.^26^ To enhance credibility, verbatim interview transcripts together with summaries of relevant narrative segments prepared by the first author were returned to all participants for member checking within two weeks after the interview. Participants were asked to provide feedback within one month, and all participants responded. Their comments were limited to minor clarifications, such as identifying the subject in narratives involving multiple individuals or providing contextual information to improve clarity. These comments were incorporated into the final transcripts, and no comments indicated that the first author had misunderstood or misrepresented the participants' intended meanings. Quotations were translated from Japanese into English by the first author. The translated quotations were refined through professional English-language editing and subsequently reviewed by the last author against the original Japanese quotations to confirm that the intended meanings had been preserved. To enhance transferability, we provided a detailed description of the study situation, participants, settings, and the collaborative practices identified. To enhance dependability, we comprehensively documented the research process. To enhance confirmability, we maintained detailed analytical worksheets and memos and kept an audit trail to record all analytic decisions.

## Results

### Participant characteristics

A total of 21 participants were interviewed, consisting of 11 hospital nurses and 10 home-visiting nurses (Table 1). Ten participants held specialized nursing certifications. The most common certification was Certified Nurse Specialist in Oncology Nursing (*n* = 5), followed by Certified Nurse in Dementia Nursing (*n* = 2), Certified Nurse in Palliative Care (*n* = 2), Certified Nurse Specialist in Home-Care Nursing (*n* = 1), Certified Nurse in Breast Cancer Nursing (*n* = 1), and Certified Nurse in Cancer Chemotherapy Nursing (*n* = 1). Two participants held multiple certifications.

**Table 1:** Participant characteristics.

Characteristics	*n*
**Age group, years**	
30s	4
40s	10
50s	6
60s	1
**Gender**	
Male	3
Female	18
**Specialized certification**^a^	
Oncology nursing (CNS)	5
Home-care nursing (CNS)	1
Dementia nursing (CN)	2
Palliative care (CN)	2
Cancer chemotherapy nursing (CN)	1
Breast cancer nursing (CN)	1
Registered nurses without advanced certification	11
**Number of participating facilities (number of participants)**^b^	
Designated cancer care hospital	2 (3)
General cancer treatment hospital	7 (8)
Home-visiting nursing station	8 (10)
**Clinical experience, years**	
10–20	8
21–30	8
31–40	4
> 40	1
**Number of people with cancer and dementia cared for per nurse per year**	
1–10	11
11–20	4
21–30	3
> 30	3

CN, Certified Nurse; CNS, Certified Nurse Specialist.

a Count may exceed the number of individuals. These certifications are advanced clinical nursing credentials granted by the Japanese Nursing Association.

b Some participants were from the same facility.

### Collaborative nursing practices supporting home-based living for people living with cancer and dementia

Six categories and 22 subcategories were generated from the analysis (Table 2).

**Table 2:** Categories, subcategories, and concepts for the theme of collaborative nursing practices supporting home-based living for people living with cancer and dementia.

Category	Subcategory	Concepts
Team building for high-quality geriatric oncology nursing	Integrating expertise of cancer care and dementia care specialists	Cancer care and dementia care specialists collaborate to provide care
	Identifying key nurses	Identifying nurses capable of taking leadership in caring for patients with both cancer and dementia
		Identifying nurses who can understand patients particularly deeply
		Identifying nurses who can serve as the hub for communication across care settings
	Bridging hospital and home-care perspectives to improve nursing quality	Bridging hospital and home-care perspectives by letting go of fixed assumptions to improve nursing quality
		Bridging the gap between medical and long-term care to enhance care quality
	Enhancing nurses' flexibility in providing dementia care	Developing nurses capable of flexible engagement with dementia
		Even if not remembered later, cherishing that moment of reassurance
		Providing care with an understanding of dementia types
		Raising the baseline quality of care
Reconstructing the patient's intention	Stabilizing unstable decision-making under concurrent illness	Addressing nurses' dilemmas regarding aggressive cancer treatment for people with dementia
		Ensuring the psychological safety of surrogate decision-makers
		Identifying individuals involved in decision-making
		Understanding the structure of conflicts arising from differing values between hospitals and home-care settings
		Creating opportunities to discuss the patient's future
	Visualizing the trade-offs between cancer care and daily life	Accurately assessing the state of dementia and treatment risks
		Clarifying and distinguishing medical and daily living challenges
		Recognizing the financial burden of cancer treatment for patients requiring dementia care
		Avoiding withholding cancer treatment solely due to dementia
		Using tools such as decision aids
	Interpreting incompletely expressed needs from multiple perspectives	Sharing nurses' understandings of what matters most to the patient
		Respecting refusal of cancer treatment as a patient's own choice
		Fostering environments and relationships that support self-expression in patients with dementia
		Inferring the unspoken feelings and intentions of patients
		Serving as an advocate for patients and families with other health care professionals
	Identifying the limits of cancer treatment and resetting the direction of care goals	Recognizing signs of disease progression and engaging in repeated discussions about when to discontinue treatment
		Predicting prognosis from both dementia and cancer perspectives
		Supporting patient- and family-centered decision-making about discontinuation of treatment
Reorganizing the care system to sustain home-based living	Promoting medical, dental, and long-term care collaboration to support vulnerabilities	Establishing opportunities for relevant health care professionals to meet face-to-face simultaneously
		Communicating nursing judgments to multidisciplinary teams
		Promoting oral and dental care during cancer treatment
		Identifying each profession's scope of practice
		Establishing supports aligned with the reality of caregiving between older adults
		Promoting support in preparation for unexpected situations such as disasters
		Creating an environment where patients feel more comfortable accepting home-care professionals
		Connecting patients with cancer and dementia to multiple appropriate professionals
		Establishing smaller forms of support within the framework of community-based integrated care
		Building grassroots connections rather than relying solely on institutional systems
	Ensuring a safety net	Arranging hospital admission as a safety net in case of emergencies
		Securing support from both medical and administrative perspectives
	Establishing a timely consultation system without occupational barriers	Establishing a consultation system involving multidisciplinary teams and nurses, considering diverse facility backgrounds
		Maintaining a system that facilitates easy consultation even after patient discharge
		Creating a system to respond promptly to consultation needs
	Preventing scattered and fragmented information	Sharing treatment history as a key resource for coordinated care
		Utilizing information from informal sources
		Creating an environment where information flows easily from other professionals and departments
		Establishing systems to ensure necessary information is shared
		Exploring ways to maximize the use of their own information
		Placing professionals capable of medical judgment in key positions where information converges
Anticipating interactions between cancer- and dementia-related symptoms and sharing management strategies	Caring for symptoms that patients cannot verbalize	Inferring symptoms that cannot be verbalized from behavior and facial expressions
		Evaluating symptoms while being mindful that phrasing questions can alter patient responses
		Addressing anxiety arising from a lack of understanding about symptoms
		Using family information about symptom trajectory to facilitate a comprehensive assessment
	Tailoring symptom management to patients' abilities, values, and home life	Exploring symptom management strategies centered on home-based living
		Exploring pain relief methods that do not rely on rescue doses
		Considering symptom management strategies that reduce family caregiving burdens
		Proposing symptom management strategies aligned with the individual's values
		Avoiding general assumptions about patients' inability to manage their symptoms and acknowledging that each person manages them according to their own understanding
		Clearly defining the scope of medical procedures and nursing care available at home
		Enhancing cancer symptom management skills among long-term care staff during cancer treatment
	Predicting the impact of symptoms arising from cancer treatments on dementia	Predicting symptoms associated with rapidly diversifying anticancer drugs and implementing preventive measures
Preventing or mitigating functional decline with disease progression	Sharing patients' ADL status based on objective indicators and assessments	Sharing ADL status across different care settings
		Screening baseline ADL before cancer treatment begins
		Assessing patients' true ADL capabilities using a valid assessment tool
	Developing rehabilitation plans to safeguard daily living	Reflecting the results of the home assessment in rehabilitation goals
		Developing rehabilitation plans to ensure the safety of patients with bone metastases and paralysis
		Establishing environmental and social resources to enable increased activity levels
		Supporting a balance between activity and rest
		Enhancing motivation for daily activities
	Stabilizing BPSD	Creating an environment reflecting the patient's lifestyle and preferences
		Reducing the use of invasive catheters
		Avoiding physical restraints
		Integrating humanistic care, supporting patients through compassionate care
		Using language and interactions that do not make patients feel threatened
		Preventing complications related to wandering
Caring for families experiencing dementia-related ambiguous loss and cancer-related losses	Facilitating acceptance of external support	Helping families provide care safely using available resources
		Proposing alternatives to reduce caregiving burdens
		Coordinating respite hospitalizations
		Ensuring access to a community support network
		Building relationships starting when families need support
	Promoting psychological preparedness for the dual losses	Supporting families to feel fulfilled in caregiving while coping with dual losses
		Supporting surrogate decision-making regarding sedation and pain relief
		Supporting families through sudden changes and frailty
		Supporting traditional family relationships beyond caregiving roles
	Clarifying family caregiving limits	Assessing the caregiving burden through family members' attitudes toward the patient
		Assessing family understanding to identify confusion or burden
		Determining whether the family desires opportunities for rest
		Understanding the family's “signs of reaching their limit”
	Supporting families by ensuring continuity of cancer care	Ensuring emergency admission when needed
		Preventing denial of necessary inpatient care due to dementia

ADL, activities of daily living; BPSD, behavioral and psychological symptoms of dementia.

The core category, “Recalibrating life continuity while living with cancer and dementia,” represented the central process underlying all categories (Fig. 1).

**Fig. 1:**
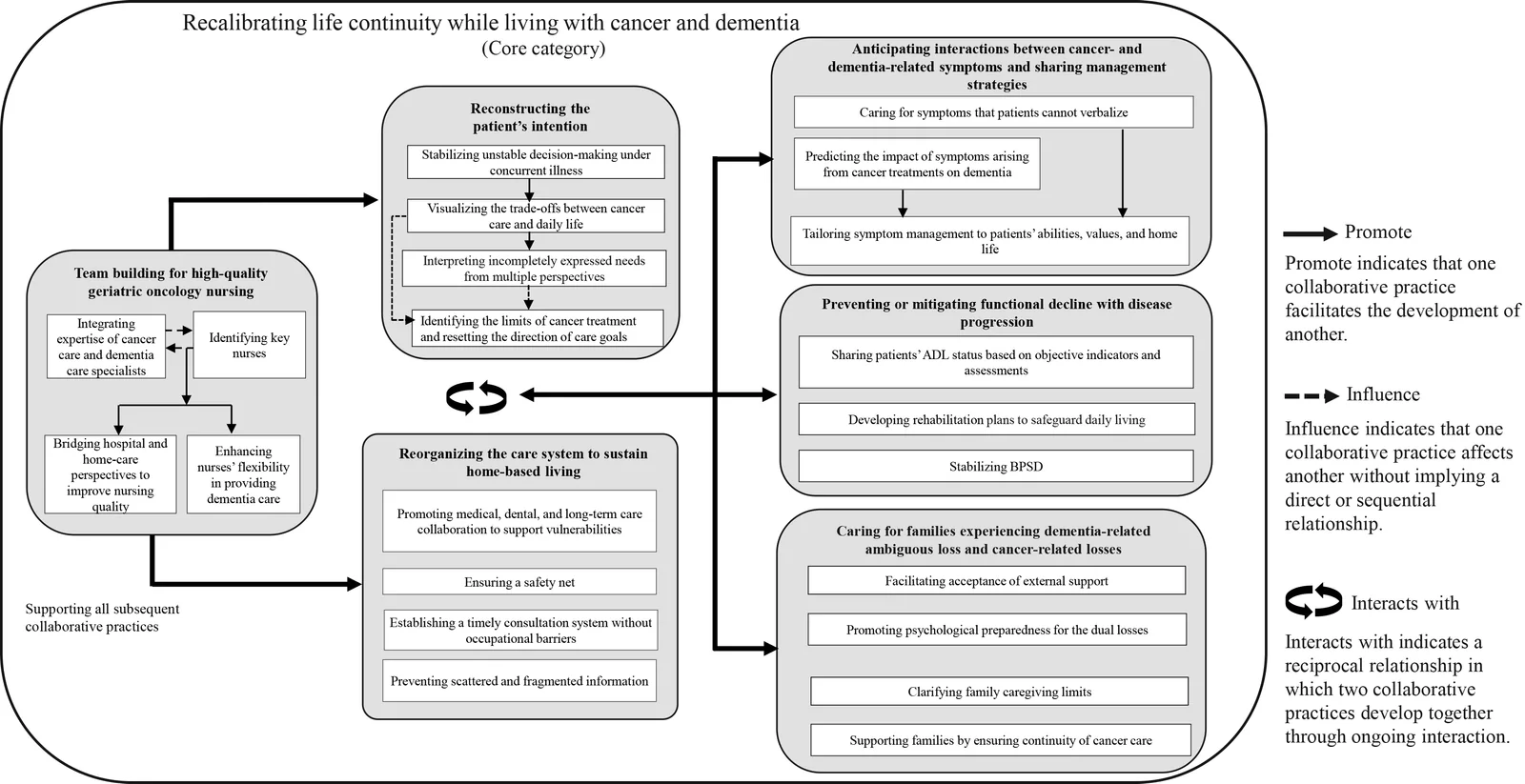
Conceptual model of collaborative nursing practices supporting home-based living for people living with cancer and dementia The relationships shown represent interpretations developed through cross-case analysis of nurses' accounts; they were not directly observed or tested in this study. ADL, activities of daily living; BPSD, behavioral and psychological symptoms of dementia.

### Storyline of the theoretical model

The relationships among categories in the theoretical model were developed through cross-case analysis of individual nurses' accounts. Collaborative nursing practices began with “Team building for high-quality geriatric oncology nursing.” Through “Integrating expertise of cancer care and dementia care specialists” and “Identifying key nurses,” nurses expanded their collaborative practice by “Bridging hospital and home-care perspectives to improve nursing quality” and “Enhancing nurses’ flexibility in providing dementia care.”

“Team building for high-quality geriatric oncology nursing” promoted the subsequent collaborative practices. Building on this foundation, nurses engaged in “Reconstructing the patient's intention” by “Stabilizing unstable decision-making under concurrent illness,” “Visualizing the trade-offs between cancer care and daily life,” and “Interpreting incompletely expressed needs from multiple perspectives.” These practices sometimes prompted nurses to engage in “Identifying the limits of cancer treatment and resetting the direction of care goals.”

“Reconstructing the patient's intention” and “Reorganizing the care system to sustain home-based living” were continuously and reciprocally linked. As nurses developed a clearer understanding of the preferences and priorities of people living with cancer and dementia, they reorganized the care system to address emerging needs and vulnerabilities, which, in turn, enabled further discussion of patients' priorities and ongoing interpretation of their intentions. Nurses addressed these vulnerabilities by “Promoting medical, dental, and long-term care collaboration to support vulnerabilities,” “Ensuring a safety net,” “Establishing a timely consultation system without occupational barriers,” and “Preventing scattered and fragmented information.”

“Reconstructing the patient's intention” and “Reorganizing the care system to sustain home-based living” together promoted subsequent collaborative practices, including “Anticipating interactions between cancer- and dementia-related symptoms and sharing management strategies,” “Preventing or mitigating functional decline with disease progression,” and “Caring for families experiencing dementia-related ambiguous loss and cancer-related losses.” These categories did not occur in a fixed sequence. Nurses engaged in “Anticipating interactions between cancer- and dementia-related symptoms and sharing management strategies” by first “Caring for symptoms that patients cannot verbalize,” then “Predicting the impact of symptoms arising from cancer treatments on dementia,” and ultimately “Tailoring symptom management to patients' abilities, values, and home life.”

Nurses also engaged in “Preventing or mitigating functional decline with disease progression.” They collaborated with multidisciplinary professionals by “Sharing patients’ activities of daily living (ADL) status based on objective indicators and assessments,” “Developing rehabilitation plans to safeguard daily living,” and “Stabilizing BPSD” to maintain their functional abilities and support continued home-based living.

Concurrently, nurses engaged in “Caring for families experiencing dementia-related ambiguous loss and cancer-related losses.” Families were required to navigate not only the gradual, ambiguous losses associated with dementia but also the additional uncertainty and losses caused by cancer progression. In response, nurses focused on “Facilitating acceptance of external support,” “Promoting psychological preparedness for the dual losses,” “Clarifying family caregiving limits,” and “Supporting families by ensuring continuity of cancer care.”

These practices constituted an ongoing process of “Recalibrating life continuity while living with cancer and dementia.”

### Team building for high-quality geriatric oncology nursing

This category comprised four interconnected subcategories that established the collaborative foundation for supporting home-based living.

“Integrating expertise of cancer care and dementia care specialists.” Nurses integrated expertise in cancer and dementia care.

When caring for patients with dementia, I know that people with dementia still hold on to the worldview and the life-shaped maturity they've built over the years, and that we can use those parts of them in their everyday care. I realize that I can't really address that kind of support based solely on my expertise in cancer care. So, I consult with certified nurses in dementia care working in home-care or long-term care facilities, and we work together to determine the best approach to care (ID-I, hospital nurse).

“Identifying key nurses.” Nurses identified key nurses who coordinated communication across professionals and care settings.

I think strategically about who to contact first and who can best connect us to ensure the patient's care proceeds smoothly. I look for the key nurse because once I consult that person, they can tell me who to involve and help connect me with the right people (ID-D, home-visiting nurse).

“Bridging hospital and home-care perspectives to improve nursing quality.” To bridge these perspectives, nurses let go of fixed assumptions and developed a shared understanding of people living with cancer and dementia.

Sometimes I feel that the values prioritized by home-visiting nurses differ from those prioritized by hospital nurses focused on cancer treatment. For example, if it is stage I colon or gastric cancer, surgery can be curative and the hospitalization may only be about a week. From that perspective, I tend to think that surgery might be a good option because the patient with dementia could maintain their own quality of life afterwards. However, from the home-visiting nurses’ side, they might see it differently: the patient currently has no symptoms and is enjoying their life as it is. They may worry that hospitalization and surgery could cause delirium or a decline in. ADL In that case, they might feel it would be better not to proceed with surgery. That is why I think it is important for us to discuss these perspectives together (ID-C, hospital nurse).

“Enhancing nurses’ flexibility in providing dementia care.” Nurses enhanced their flexibility in responding to dementia-related needs through education and ongoing knowledge sharing.

We need to see situations from the patient's perspective and understand that certain behaviors are part of dementia. When care is refused, we consider how to manage those feelings and respond flexibly. We consider whether it was simply too early in the morning for a patient with dementia, or whether the involvement of a stranger was the problem. This is something we should incorporate into staff education (ID-P, hospital nurse).

### Reconstructing the patient's intention

This category describes nurses’ efforts to understand and interpret the preferences and intentions of people with cancer and dementia through collaborative decision-making.

“Stabilizing unstable decision-making under concurrent illness.” First, nurses stabilized unstable decision-making by identifying individuals who were able to participate in decision-making and tailoring support according to their readiness and circumstances.

When family members begin to come to terms with dementia, they realize that the person is no longer quite the same mother, father, or spouse they once knew. I assessed whether they had come to terms with that loss; in this case, since I sensed that the husband was beginning to accept his wife's dementia, I determined that he was ready to make decisions regarding her cancer treatment. If he hadn't yet come to terms with his wife's dementia, he wouldn't have been in a position to make decisions about her cancer treatment, and we would have had to consider alternative approaches rather than proceeding with surrogate decision-making with him (ID-Q, hospital nurse).

“Visualizing the trade-offs between cancer care and daily life.” Nurses then clarified the trade-offs between cancer treatment and daily life by considering how treatment would affect the future lives and priorities of people living with cancer and dementia.

Cancer treatment inevitably involves various trade-offs. Patients must accept certain exchanges, such as experiencing side effects in order to prolong life. Even when treatment is intended for symptom relief, temporary side effects may still need to be tolerated. Many patients express things like, ‘I want to at least be able to do this,’ or ‘I don't want to lose this ability.’ However, for patients with dementia, it is often difficult to verbalize these priorities. That is why nurses try to identify what cannot be compromised and what values the patient is trying to preserve (ID-A, hospital nurse).

“Interpreting incompletely expressed needs from multiple perspectives.” Through repeated discussions and careful interpretation of fragmented words and behaviors associated with cognitive decline, nurses sought to understand patients’ incompletely expressed needs from multiple perspectives.

Even when people with dementia say they do not want treatment, we do not assume they lack decision-making capacity. Because their wishes may change from day to day, we carefully listen to what they are saying at each moment. Even when they say they do not want treatment, we may realize that they also have a desire to continue it. By carefully listening to each expression in its context, we gradually uncover their underlying needs (ID-A, hospital nurse).

“Identifying the limits of cancer treatment and resetting the direction of care goals.” When the circumstances or wishes of people living with cancer and dementia changed, nurses reassessed the limits of cancer treatment and reset care goals.

In home care, we sometimes encounter patients who are no longer able to take their medication or who no longer wish to do so. In such cases, while taking the patient's wishes into account, we inform the hospital's medical team of the situation and discuss whether treatment should continue or if it is time to reassess the care goals (ID-D, home-visiting nurse).

### Reorganizing the care system to sustain home-based living

This category illustrates how nurses reorganized the care system to sustain home-based living for people living with cancer and dementia. To enable people to remain at home, they coordinated care across medical, dental, and long-term care services while considering the realities faced by aging caregivers and financial constraints. They also maintained a basic safety net, established timely consultation across professional boundaries, and continuously shared information to prevent patient-related information from becoming fragmented.

Older adults with both cancer and dementia can become frail very quickly. When their ADL begin to decline, we involve rehabilitation professionals to help maintain function. If chewing or swallowing becomes difficult, we ask speech-language therapists or dentists to become involved and help connect patients and their families with the appropriate professionals (ID-R, home-visiting nurse).

Some people with dementia are living in extreme financial hardship. We work with administrative services to ensure they receive the care they need. Sometimes a person with dementia is brought to the hospital with no one knowing who is supporting them. In those situations, we connect them with people or organizations that can help—including community resources and even the police—so that, at the very least, they are not left without support (ID-S, home-visiting nurse).

One of my roles is to continue receiving consultations even after the patient has been discharged. Sometimes we would like to refer them to the palliative care outpatient clinic, but patients or their families may feel hesitant about seeing new nurses and multiple professionals or worry that, due to dementia, the patient might not be able to go to a palliative care ward. In those situations, I receive the consultation and try to coordinate the response. Sometimes we ask the home-care professionals to come to the hospital so they can discuss the situation directly. If it is a simple matter, I can ask the doctor and relay the information. But when the physician's judgment is necessary, we arrange a small conference with the home-care professionals to discuss the case together (ID-U, hospital nurse).

Because patients with dementia often cannot tell us their treatment journey themselves, we want to know what treatments they received, how many cycles they completed, when the disease progressed, when treatment was especially difficult for them, and when they were doing well enough that they might even have been able to travel. We usually meet patients and their families for the first time at home, so it makes a real difference whether we can say, ‘You've been through so much,’ or whether we have to ask, ‘When were you diagnosed with cancer?’ A four-line handover saying, ‘Chemotherapy was ineffective, and the patient has been referred for home care,’ is completely different from understanding the whole process. That's the kind of information we ask for (ID-T, home-visiting nurse).

### Anticipating interactions between cancer- and dementia-related symptoms and sharing management strategies

This category captures how nurses collaboratively anticipated interactions between cancer- and dementia-related symptoms and developed management strategies to reduce symptom-related instability.

“Caring for symptoms that patients cannot verbalize.” First, because people with dementia were often unable to verbalize their symptoms, nurses identified subtle signs of discomfort through careful observation, repeated assessment, and information sharing with family members and other professionals.

Some patients with Alzheimer's disease are difficult to assess from their facial expressions alone. They may be unable to express where it hurts, what kind of pain they have, or when it hurts. We watch their movements—for example, where they repeatedly touch their body—and ask, ‘Does it hurt here?’ They may tell the nurse, ‘No,’ but tell a family member, ‘Yes, it hurts.’ That's why we tried to have the same nurse visit them consistently (ID-K, home-visiting nurse).

“Predicting the impact of symptoms arising from cancer treatments on dementia.” Nurses also anticipated how cancer treatment-related symptoms might worsen dementia-related symptoms.

In the case of patients with dementia, when patients say they feel unwell, the situation may already be serious. For example, they may suddenly develop a high fever, and by then it can already be too late. Because there are now many different types of cancer treatments, including molecularly targeted therapies and drugs such as pembrolizumab, we often hear that nurses need to study the specific side effects of each medication. From the hospital side, we also feel that it is important for home-visiting nurses to be familiar with these potential adverse effects. If they can notice early signs and provide feedback to the hospital, we may be able to intervene before the condition becomes severe. When we collaborate, we try to be mindful of this (ID-M, hospital nurse).

“Tailoring symptom management to patients’ abilities, values, and home life.” Nurses worked together to identify where the interaction between cancer- and dementia-related symptoms could be interrupted and tailored symptom management to ensure that it remained simple, feasible, and aligned with patients' values and home life.

I never assume that people with dementia are unable to manage their own symptoms. Instead, I carefully assess what they can and cannot do. Even after chemotherapy caused her hair to fall out, she did not seem distressed and explained it in her own way, saying, ‘Well, I'm getting older, so my hair is falling out.’ When her husband bought her a hat, she always wore it when she went out. I realized that taking care of her appearance in her own way was also a form of self-care. I started by recognizing what she was still able to do, rather than focusing on what she could not do (ID-C, hospital nurse).

### Preventing or mitigating functional decline with disease progression

To address these challenges, nurses shared the ADL status of people living with cancer and dementia using objective indicators and clinical assessments, developed rehabilitation plans tailored to their home environments and daily lives, and adapted care to their behaviors and routines to stabilize BPSD while maintaining safety.

Hospital nurses may not always be able to imagine what the patient's home environment is like. Whether a patient can return home safely depends not only on their physical function but also on objective assessments of their functional status, such as the Karnofsky Performance Status, Palliative Performance Scale, and Barthel Index, information from family members, and the risk of falls at home. We would like these factors to receive more attention when planning discharge (ID-J, home-visiting nurse).

Because the sites of bone metastasis have been identified, we always confirm together with the hospital nurses how the patient should move, for example, avoiding weight-bearing on that area or adjusting transfer techniques (ID-B, home-visiting nurse).

Basically, because the goal is for the patient to return home, we try to have them stand for about 20 minutes a day. If they can stand for 20 minutes, we believe their ADL will not decline and they will be able to manage daily life. We carefully record how long they stand each day, for example, how many seconds they stood when going to the toilet or how many minutes they spent in rehabilitation during the day, and we keep track of the total time (ID-E, hospital nurse).

Over the years, our wards have gradually shifted away from using physical restraints. When patients with dementia wander, we are more likely to walk alongside them than try to stop them, so as not to worsen their BPSD. The shared philosophy has become to stay with the patient, ensure their safety, and provide care in a way that respects who they are (ID-E, hospital nurse).

### Caring for families experiencing dementia-related ambiguous loss and cancer-related losses

This category captures nurses' collaborative efforts to support families experiencing the overlapping challenges of cancer progression and dementia while helping them cope with dementia-related ambiguous loss and cancer-related losses. Nurses facilitated acceptance of external support, promoted psychological preparedness for the dual losses, clarified the limits of family caregiving, and ensured continuity of cancer care to enable families to continue providing care with appropriate support.

When families tell me that the patient refuses to take medication, I discuss possible options with them, such as reducing the number of medications, switching treatments, or considering other approaches. I try to help families accept this support from other professionals so that they have more people to rely on. We think through the situation together, which helps reduce the family's burden (ID-D, home-visiting nurse).

When I notice something unusual in the way family members speak to the patient, such as unexpectedly harsh expressions or anger, I start paying closer attention to the family relationship. For example, when a patient with dementia becomes incontinent due to cancer-related bladder or rectal dysfunction and the family reacts with anger, I anticipate that problems may eventually arise. So, I prepare in advance by documenting symptoms carefully and considering connections with community resources (ID-P, hospital nurse).

Families have to cope with the progression of dementia, gradual age-related physical decline, and the added complexity of cancer. Symptoms may suddenly worsen, the patient's condition may change unexpectedly, and cancer treatment can bring both benefits and burdens. We need to work through all of this together with families, including the additional challenges caused by dementia. It's unrealistic to expect families to understand everything when they have just been told about the cancer at such a difficult time. The only thing we can do is walk alongside them—and if they allow us to, we can continue supporting them (ID-C, hospital nurse).

Before the next cycle of treatment, patients often return home even when they are not feeling well. We explain to families that if the patient develops a fever, has difficulty breathing, or becomes dehydrated because they cannot eat, they should not hesitate to seek help. For a mild fever, they may try antipyretics, but if the temperature exceeds 38°C or the patient's breathing worsens, we tell them to come to the emergency department at any time, even at night. During weekdays, they can also call the outpatient clinic if they are worried. Our priority is to make access to medical care as easy as possible. We reassure them that there is always a place where they can receive care and that they can return whenever they need help (ID-A, hospital nurse).

## Discussion

Three key insights emerged from the proposed model and warrant further discussion.

### Team building for high-quality geriatric oncology nursing

The category “Team building for high-quality geriatric oncology nursing” identifies team building as the foundation for subsequent collaborative nursing practices. By integrating the expertise of cancer care and dementia care specialists and bridging hospital and home-care perspectives, nurses developed a shared clinical perspective for understanding the complex needs of people living with cancer and dementia. This shared perspective enabled them to question deeply rooted assumptions, such as “patients with dementia cannot receive cancer treatment” or “cancer treatment inevitably requires sacrificing daily life.” Letting go of these implicit stereotypes enabled nurses to avoid framing care as a simple choice between prioritizing cancer treatment and preserving daily functioning. Instead, they assessed the physical, psychosocial, and social conditions of each individual. By challenging disease-centered assumptions, nurses were able to move beyond viewing dementia merely as a factor of vulnerability that complicates cancer treatment or home care. Rather, they understood dementia within the context of the individual's remaining abilities, hopes, relationships, and living circumstances. This broader perspective allowed nurses to identify opportunities for individualized care while maintaining a realistic understanding of the challenges associated with both conditions. Team building was rooted in establishing a shared philosophy of care centered on the individual, rather than on either condition alone.

Previous research has shown that nurses' attitudes toward people with dementia in designated cancer care hospitals influence the implementation of person-centered care.^23^ The present study extends this knowledge by suggesting how collaborative team building may contribute to the development of a shared understanding and its translation into clinical practice.

### Reciprocal relationship between “Reconstructing the patient's intention” and “Reorganizing the care system to sustain home-based living”

The proposed model suggests that “Reconstructing the patient's intention” and “Reorganizing the care system to sustain home-based living” were mutually reinforcing processes. “Reconstructing the patient's intention” informed the organization of care, whereas changes in available care resources often prompted nurses to revisit and reinterpret the patient's intentions. Across hospital and home-visiting nurses' accounts, this reciprocal relationship involved developing not only a shared understanding of patients' current conditions but also a shared anticipation of the likely course of both cancer and dementia. As decision-making capacity may fluctuate over time, people living with cancer and dementia may retain the capacity to make some decisions while lacking the capacity for others.^27^, ^28^ Nurses repeatedly revisited patients' preferences while reorganizing care systems to support their continued participation in decision-making. These practices can be understood as efforts to optimize modifiable elements of the decision ecology,^29^ thereby maximizing opportunities for people living with cancer and dementia to express their intentions under changing circumstances. Reorganizing the care system required nurses to establish timely consultation and collaboration across medicine, dentistry, rehabilitation, and long-term care. In doing so, nurses functioned as the hub through which information, clinical judgments, and care resources were continuously integrated and adapted to the patients' changing needs. While a previous study demonstrated the benefits of multidisciplinary interventions in cancer care,^30^ the present findings, derived from nurses' accounts, suggest that nurses may play a central role in sustaining consultation and collaboration as part of the ongoing reorganization of care systems throughout home-based care.

### Supporting continuity of home-based living through symptom management, functional support, and family care

The proposed model further suggests that the categories of “Anticipating interactions between cancer- and dementia-related symptoms and sharing management strategies,” “Preventing or mitigating functional decline with disease progression,” and “Caring for families experiencing dementia-related ambiguous loss and cancer-related losses” represent three complementary collaborative nursing practices.

Regarding symptom management and maintenance of physical function, hospital nurses did not assume that people living with cancer and dementia were stable simply because they appeared functional during hospital visits. They recognized that those who seemed momentarily active in the hospital might still be experiencing falls or functional decline at home. Consequently, hospital nurses deliberately sought information from home-visiting nurses to understand their daily lives, anticipate emerging problems, and adapt care before these changes threatened continued home-based living. Anticipatory symptom management involved efforts to identify non-verbal signs of distress, an approach that was particularly important for people living with cancer and dementia.

Notably, participants did not describe the use of standardized symptom assessment tools in their accounts. Although several validated instruments exist for detecting non-verbal or poorly articulated distress in people with dementia,^31^, ^32^ no consensus has been established regarding which tool should guide assessment for people with both cancer and dementia.^33^ This may indicate a need for a shared clinical framework for symptom assessment across hospital and home-care settings. Similar variation was reflected in participants’ accounts of physical function assessment; for example, while one hospital used a clear benchmark for activity (e.g., maintaining 20 minutes of standing), comparable criteria were not described across other hospitals or home-care settings. These accounts suggest potential value in a shared approach to assessing symptoms and physical function across care settings, alongside information sharing. Developing an evidence base and shared assessment guidelines for geriatric oncology nursing may therefore strengthen the consistency and continuity of care for people with both cancer and dementia.

### Implications for nursing practice

The proposed model provides a framework for understanding collaborative nursing practices across hospital and home-care settings. Educational programs can use this model to help nurses understand the relationships among collaborative practices, rather than teaching individual competencies in isolation. The findings further suggest the potential importance of ongoing case-based communication between hospital and home-visiting nurses. Sharing clinical information alone may be insufficient, highlighting the need for opportunities to jointly interpret the changing conditions of people living with cancer and dementia and reconsider care plans to support continued home-based living. By making the relationships among collaborative practices explicit, the model may help teams identify aspects of collaboration that require strengthening and anticipate how improvements in one area may enhance collaborative nursing practices as a whole.

### Limitations

This study has certain limitations. First, recruitment through professional networks may have resulted in the inclusion of nurses who were particularly interested in or positively oriented toward collaborative practice, and the findings may therefore not fully reflect the diversity of perspectives in clinical settings. Second, the participants had substantial clinical experience, and nearly half held specialized certifications. Their perspectives may differ from those of nurses with less clinical experience, which should be considered when assessing the transferability of the findings to broader nursing populations. Third, the findings were generated within the Japanese health care and long-term care systems, and their transferability to settings with different service structures, professional roles, and available resources should therefore be considered with caution. Fourth, the findings were based exclusively on nurses’ accounts and were not examined from the perspectives of patients, families, or other health care and long-term care professionals. In addition, the study did not directly observe collaborative interactions or examine accounts from hospital and home-visiting nurses involved in the same cases. Therefore, these limitations should be considered when interpreting the findings and the proposed model.

## Conclusions

This study identified the core category, “Recalibrating life continuity while living with cancer and dementia,” and six categories of collaborative nursing practices supporting home-based living for people living with cancer and dementia. “Team building for high-quality geriatric oncology nursing” provided the foundation for subsequent collaborative nursing practices. By integrating expertise across care settings and disciplines, nurses collaboratively engaged in “Reconstructing the patient's intention,” “Reorganizing the care system to sustain home-based living,” “Anticipating interactions between cancer- and dementia-related symptoms and sharing management strategies,” “Preventing or mitigating functional decline with disease progression,” and “Caring for families experiencing dementia-related ambiguous loss and cancer-related losses.” Future research is needed to determine the extent to which these practices are implemented across diverse clinical settings and clarify their impact on outcomes for people living with cancer and dementia and their families.

## Ethics statement

This study was approved by the Ethics Review Committee of the Graduate School of Nursing, Osaka Metropolitan University (Approval No. 2024–74; March 14, 2025). The study procedures complied with the ethical principles of the Declaration of Helsinki (2024) and with all relevant laws and institutional guidelines of Osaka Metropolitan University. Written informed consent was obtained from all participants.

## CRediT authorship contribution statement

**Ayumi Takao**: Conceptualization, Methodology, Investigation, Data Curation, Formal Analysis, Writing – Original Draft, Funding Acquisition.

**Ikumi Iida:** Supervision, Writing – Review & Editing.

**Hiroko Sakuma:** Formal Analysis, Supervision, Writing – Review & Editing.

**Hiromi Sakuda:** Supervision, Validation, Writing – Review & Editing.

All authors have read and approved the final manuscript.

## Data availability

The data that support the findings of this study are available from the corresponding author upon reasonable request.

## Declaration of generative AI and AI-assisted technologies in the writing process

No generative AI or AI-assisted technologies were used in the preparation of this work.

## Funding

This work was supported in part by the Foundation for Promotion of Cancer Research in Japan and 10.13039/501100001691JSPS KAKENHI (Grant No. 25K20714). The funders had no role in the study design; data collection, analysis, or interpretation; preparation of the manuscript; or the decision to submit the article for publication.

## Conflict of interest statement

The authors declare no conflict of interest.
