# Supplementary material for: Collaborative nursing practices supporting home-based living for people living with cancer and dementia: A modified grounded theory study
Source: Asia Pac J Oncol Nurs. 2026 Oct 17;13:101048. doi: 10.1016/j.apjon.2026.101048 (PMC13744370; doi:10.1016/j.apjon.2026.101048)
Supplement: Supplementary file 1 [file mmc1.docx]

Supplementary Table S1. Interview guide

| **Topics** | **Example interview questions** |
| --- | --- |
| Challenges experienced by people with cancer and dementia and their families | ・What kinds of difficulties did people with cancer and dementia and their families experience in continuing home-based care?  ・Could you describe a memorable case? |
| Nursing care provided | ・How did you support people with cancer and dementia and their families in continuing home-based care?  ・What did you consider important in providing this support? |
| Collaborative practice | ・How did you collaborate with hospital nurses or home-visiting nurses?  ・What facilitated or hindered this collaboration? |
| Probing questions | ・Could you tell me more about that?  ・Could you give a specific example? |
